# Cumulative 6-Year Risk of Screen-Detected Ductal Carcinoma In Situ by Screening Frequency

**DOI:** 10.1001/jamanetworkopen.2023.0166

**Published:** 2023-02-20

**Authors:** Brian L. Sprague, Shuai Chen, Diana L. Miglioretti, Charlotte C. Gard, Jeffrey A. Tice, Rebecca A. Hubbard, Erin J. Aiello Bowles, Peter A. Kaufman, Karla Kerlikowske

**Affiliations:** 1 Office of Health Promotion Research, University of Vermont, Burlington; 2 Department of Surgery, University of Vermont, Burlington; 3 University of Vermont Cancer Center, Burlington; 4 Division of Biostatistics, Department of Public Health Sciences, University of California, Davis; 5 Kaiser Permanente Washington Health Research Institute, Kaiser Permanente Washington, Seattle; 6 Department of Economics, Applied Statistics, and International Business, New Mexico State University, Las Cruces; 7 Division of General Internal Medicine, Department of Medicine, University of California, San Francisco; 8 Department of Biostatistics, Epidemiology, and Informatics, University of Pennsylvania Perelman School of Medicine, Philadelphia; 9 Division of Hematology/Oncology, University of Vermont Cancer Center, Burlington; 10 Department of Medicine, University of California, San Francisco; 11 Department of Epidemiology and Biostatistics, University of California, San Francisco; 12 General Internal Medicine Section, Department of Veterans Affairs, University of California, San Francisco

## Abstract

**Importance:**

Detection of ductal carcinoma in situ (DCIS) by mammography screening is a controversial outcome with potential benefits and harms. The association of mammography screening interval and woman’s risk factors with the likelihood of DCIS detection after multiple screening rounds is poorly understood.

**Objective:**

To develop a 6-year risk prediction model for screen-detected DCIS according to mammography screening interval and women’s risk factors.

**Design, Setting, and Participants:**

This Breast Cancer Surveillance Consortium cohort study assessed women aged 40 to 74 years undergoing mammography screening (digital mammography or digital breast tomosynthesis) from January 1, 2005, to December 31, 2020, at breast imaging facilities within 6 geographically diverse registries of the consortium. Data were analyzed between February and June 2022.

**Exposures:**

Screening interval (annual, biennial, or triennial), age, menopausal status, race and ethnicity, family history of breast cancer, benign breast biopsy history, breast density, body mass index, age at first birth, and false-positive mammography history.

**Main Outcomes and Measures:**

Screen-detected DCIS defined as a DCIS diagnosis within 12 months after a positive screening mammography result, with no concurrent invasive disease.

**Results:**

A total of 916 931 women (median [IQR] age at baseline, 54 [46-62] years; 12% Asian, 9% Black, 5% Hispanic/Latina, 69% White, 2% other or multiple races, and 4% missing) met the eligibility criteria, with 3757 screen-detected DCIS diagnoses. Screening round–specific risk estimates from multivariable logistic regression were well calibrated (expected-observed ratio, 1.00; 95% CI, 0.97-1.03) with a cross-validated area under the receiver operating characteristic curve of 0.639 (95% CI, 0.630-0.648). Cumulative 6-year risk of screen-detected DCIS estimated from screening round–specific risk estimates, accounting for competing risks of death and invasive cancer, varied widely by all included risk factors. Cumulative 6-year screen-detected DCIS risk increased with age and shorter screening interval. Among women aged 40 to 49 years, the mean 6-year screen-detected DCIS risk was 0.30% (IQR, 0.21%-0.37%) for annual screening, 0.21% (IQR, 0.14%-0.26%) for biennial screening, and 0.17% (IQR, 0.12%-0.22%) for triennial screening. Among women aged 70 to 74 years, the mean cumulative risks were 0.58% (IQR, 0.41%-0.69%) after 6 annual screens, 0.40% (IQR, 0.28%-0.48%) for 3 biennial screens, and 0.33% (IQR, 0.23%-0.39%) after 2 triennial screens.

**Conclusions and Relevance:**

In this cohort study, 6-year screen-detected DCIS risk was higher with annual screening compared with biennial or triennial screening intervals. Estimates from the prediction model, along with risk estimates of other screening benefits and harms, could help inform policy makers’ discussions of screening strategies.

## Key Points

**Question:**

Does cumulative risk of screen-detected ductal carcinoma in situ (DCIS) vary according to mammography screening interval and clinical risk factors?

**Findings:**

For this cohort study, a well-calibrated model was developed to predict cumulative 6-year risk of screen-detected DCIS in 916 931 women. Compared with women undergoing biennial mammography, those undergoing annual mammography had a 40% to 45% higher 6-year cumulative risk of screen-detected DCIS, whereas those undergoing triennial mammography had lower risk.

**Meaning:**

This risk model provides estimates of the 6-year probability of screen-detected DCIS and can inform discussions of screening benefits and harms for those considering a screening interval other than biennial.

## Teaser

This cohort study of US women aged 40 to 74 years undergoing mammography screening uses a risk prediction model to determine the cumulative 6-year risk of screen-detected ductal carcinoma in situ.

## Introduction

Detection of ductal carcinoma in situ (DCIS) is a controversial outcome of mammography screening. The incidence of DCIS increased markedly in the US with the widespread adoption of screening mammography,^1,2^ and more than 30% of screen-detected breast cancers are DCIS.^3^ Because DCIS is a nonobligate precursor to invasive breast cancer, the detection and treatment of DCIS may reduce the risk of subsequent invasive disease,^4,5^ yet there is concern that a substantial fraction of DCIS may never lead to invasive cancer if left untreated.^2,6,7^ Overdiagnosis is challenging to estimate^8,9^ but has influenced national breast cancer screening recommendations as a potential harm of breast cancer screening.^10,11^

The US Preventive Services Task Force and American Cancer Society recommendations include elements of individual informed decision-making regarding breast cancer screening strategies, including whether to start screening before the age of 50 years and whether screens should be performed annually or biennially. Aggregate data on mammography screening benefits and harms^7,12,13^ and individual-level breast cancer risk prediction models^14^ are available to inform these decisions, yet few models provide individual-level predictions of mammography screening outcomes. Models were recently published for cumulative 6-year risk of advanced (prognostic stage II or higher) breast cancer and cumulative 10-year risk of a false-positive mammography result based on mammography screening frequency and readily available clinical risk factors.^15,16^ Prediction models for screen-detected DCIS would further inform screening decisions and guidelines.

The purpose of this study is to examine DCIS detection rates according to mammography screening interval and clinical risk factors and develop a risk prediction model to estimate the cumulative 6-year risk of screen-detected DCIS. We used a 6-year horizon to enable comparison of outcomes for 6 annual, 3 biennial, and 2 triennial screening rounds.

## Methods

### Study Setting

For this cohort study, we used observational clinical data from 6 breast imaging registries within the Breast Cancer Surveillance Consortium (BCSC): the Carolina Mammography Registry, the Kaiser Permanente Washington Registry, the New Hampshire Mammography Network, the Vermont Breast Cancer Surveillance System, the San Francisco Mammography Registry, and the Metropolitan Chicago Breast Cancer Registry. Each registry prospectively collects clinical data on women undergoing breast imaging from participating radiology facilities within its catchment area. The registries and a central statistical coordinating center received institutional review board approval from their respective institutions for active or passive consenting processes or a waiver of consent to enroll participants, link data, and perform analyses. Identifiable data are collected by each registry. Limited data sets (containing dates and residential zip codes but no other direct identifiers) are sent to the BCSC Statistical Coordinating Center for pooling and statistical analysis. All procedures were Health Insurance Portability and Accountability Act compliant, and registries and the statistical coordinating center received a federal certificate of confidentiality for the identities of women, physicians, and facilities. The study followed Strengthening the Reporting of Observational Studies in Epidemiology (STROBE) guidelines^17^ for reporting results from cohort studies and Transparent Reporting of a Multivariable Prediction Model for Individual Prognosis or Diagnosis (TRIPOD) reporting guidelines for development of the risk prediction model.

### Study Population

Women aged 40 to 74 years undergoing mammography screening (digital mammography or digital breast tomosynthesis) from January 1, 2005, to December 31, 2020, were eligible for inclusion. We excluded women with a prior history of breast cancer (invasive or DCIS), lobular carcinoma in situ, or mastectomy. Screening mammograms were identified based on the radiologist’s clinical indication for the examination. To reflect women who were routinely screened and evaluate the screening interval, we restricted the study to screening mammograms among women who underwent mammography within the prior 42 months (corresponding to the upper limit of our triennial screening interval definition). Thus, a woman’s first mammogram was not included. We also excluded mammography screening that was unilateral, was preceded by mammography within the prior 9 months, was followed by screening ultrasonography within 3 months, or occurred 12 months before or after screening magnetic resonance imaging. At least 1 year of follow-up for complete capture of cancer diagnoses was required.

### Data Collection

Participating radiology facilities provide imaging modality, examination indication, breast density, and assessment data to BCSC registries using standard nomenclature from the Breast Imaging Reporting and Data System (BI-RADS).^18^ Demographic and risk factor information is self-reported or extracted from electronic medical records. The BCSC registries ascertain breast cancer diagnoses and tumor characteristics by linking women to pathology databases; regional Surveillance, Epidemiology, and End Results programs; and state tumor registries. Deaths are obtained by linking to state death records.

### Outcome and Predictor Definitions

Screen-detected DCIS was defined as a DCIS diagnosis within 12 months after a screening mammogram with a positive final assessment (BI-RADS category 3, 4, or 5), with no invasive breast cancer diagnosis.^12^ We evaluated rates of screen-detected DCIS in relation to mammography screening interval, mammography screening modality (digital mammography vs digital breast tomosynthesis [DBT]), and 9 clinical breast cancer risk factors: age, menopausal status, first-degree family history of breast cancer, history of benign breast biopsy, BI-RADS breast density,^18^ body mass index (BMI; calculated as weight in kilograms divided by height in meters squared), age at first birth, history of false-positive screening mammography results in the previous 5 years, and race and ethnicity. Screening interval for each mammogram was defined based on the time since the woman’s prior mammogram (annual: 11-18 months; biennial: 19-30 months; and triennial: 31-42 months). Breast density is categorized by radiologists during clinical interpretation as almost entirely fatty, scattered fibroglandular densities, heterogeneously dense, or extremely dense.^18^ Postmenopausal women were those with both ovaries removed, in whom menstruation had stopped naturally, who were currently receiving postmenopausal hormone therapy, or who were 60 years or older. Premenopausal women were those who reported menstruating within the last 180 days, who used oral contraceptives, or who were younger than 45 years. History of benign breast biopsy was defined based on diagnoses abstracted from clinical pathology reports. We grouped prior benign diagnoses based on the highest grade as proliferative with atypia greater than proliferative without atypia greater than nonproliferative using published taxonomy^19,20,21,22^ or as unknown if a woman reported a prior biopsy with no available BCSC pathology result. Self-reported race and ethnicity were included as a social construct that could potentially capture differences in screen-detected DCIS risk due to social determinants of health, including inequities in access to high-quality screening and diagnostic services, and were categorized as Hispanic/Latina and for non-Hispanic/Latina as Asian, Black, White, or other or multiple races (including American Indian or Alaska Native, Native Hawaiian or Pacific Islander, and self-reported other race).

### Statistical Analysis

Analyses were conducted between February and June 2022. The screening mammogram was the unit of analysis. We estimated absolute screen-detected DCIS risk after 1 round of screening using multivariable logistic regression, including screening interval, modality, age (linear and quadratic, centered at 55 years), calendar year of screen (linear and quadratic, centered at January 31, 2020), menopausal status, first-degree breast cancer family history, benign biopsy history, BMI (categorical), breast density, age at first live birth (categorical), prior false-positive mammography result, and race and ethnicity. Before model fitting, 20 imputed values for each missing variable were generated using multiple imputation via chained equations (eMethods and eTable 5 in Supplement 1).^23^ For each covariate combination, risk scores from a single screening round were estimated by averaging over the 20 risk scores estimated in fitted logistic regression models from each imputed data set. We evaluated interactions of risk factors with age, age squared, and menopausal status and retained those that were statistically significant at a 2-sided *P* < .05 on type 3 tests; these interactions included those between linear age and BMI, linear age and prior false-positive mammography results, and menopausal status and BMI. We also tested interactions between each risk factor and screening interval; none were significant at *P* < .05 and thus were not included in the model. Mammography modality (digital mammography vs DBT) was not associated with DCIS detection and was omitted from the final model. Model calibration was estimated as the ratio of expected to observed number (E/O ratio) of screen-detected DCIS, both overall and within predicted risk decile groups. Model discriminatory accuracy was summarized using the area under the receiver operating characteristic curve (AUC). To internally validate the model, we compared the AUC from the model fit using the full data to the AUC from a model fit using 5-fold cross-validation, and the difference between them (optimism) was 0.004. To account for this small overfitting, the AUC and 95% CI were adjusted by subtracting the optimism from the estimates obtained from the full data.

The cumulative screen-detected DCIS risks after hypothetical repeat screening patterns consisting of 6 annual, 3 biennial, or 2 triennial screens occurring at 12-, 24-, or 36-month intervals, respectively, were estimated using a discrete-time survival model based on the fitted logistic regression models for 1 round of screening while accounting for competing risks of death or invasive cancer within 1 year after annual screening, 2 years after biennial screening and 3 years after triennial screening.^24^ A 6-year horizon enables comparison of outcomes for 6 annual, 3 biennial, or 2 triennial screening rounds. Mean predicted 6-year cumulative risks and IQRs for different screening intervals were estimated in a standardized population; the weights of the study population were adjusted to reflect the US female population based on age, race and ethnicity, and family history of breast cancer.^25,26^ The cumulative 6-year risk of screen-detected DCIS was categorized into 5 risk levels (high, >95th percentile; intermediate, 75th-95th percentile; average, 25th-75th percentile; low, 5th-25th percentile; and very low, ≤5th percentile) adjusted by US population weights and standardized to the same population for different screening intervals. Data were analyzed using R software, version 4.0.4 (R Foundation for Statistical Computing) and SAS software, version 9.4 (SAS Institute Inc). Two-sided α = .05 was used to determine statistical significance. The eMethods in Supplement 1 provide additional statistical methods details.

## Results

A total of 2 320 016 annual, 681 983 biennial, and 199 058 triennial mammograms in 916 931 women (median [IQR] age at baseline, 54 [46-62] years) were included, with 3757 screen-detected DCIS diagnoses. Overall, the distribution of self-reported race and ethnicity was 12% Asian, 9% Black, 5% Hispanic/Latina, 69% White, 2% other or multiple races, and 4% missing. The screening interval was shorter among women who were older, who were White, and who had a first-degree family history of breast cancer, prior benign biopsy, normal BMI, or history of false-positive mammography results (Table 1).

**Table 1.** Examination-Level Characteristics of Women Undergoing Screening Mammography by Screening Interval, Breast Cancer Surveillance Consortium, 2005-2020

Characteristic	No. (%) of examinations^a^		
	Annual (n = 2 320 016)	Biennial (n = 681 983)	Triennial (n = 199 058)
Age group, y			
40-49	550 151 (26.4)	163 440 (26.3)	58 582 (31.8)
50-59	805 860 (38.7)	249 409 (40.1)	74 274 (40.3)
60-69	724 085 (34.8)	209 137 (33.6)	51 577 (28.0)
70-74	239 920 (10.3)	59 997 (8.8)	14 625 (7.3)
Race and ethnicity			
Asian	234 941 (10.5)	108 599 (16.4)	26 190 (13.7)
Black	209 025 (9.4)	58 158 (8.8)	19 917 (10.4)
Hispanic/Latina	109 559 (4.9)	46 510 (7.0)	13 130 (6.9)
White	1 640 900 (73.5)	430 314 (65.2)	127 001 (66.4)
Other or multiple races^b^	38 421 (1.7)	16 774 (2.5)	5113 (2.7)
Missing	87 170 (3.8)	21 628 (3.2)	7707 (3.9)
Menopausal status			
Premenopausal	546 510 (28.6)	164 582 (29.2)	57 340 (35.8)
Postmenopausal	1 362 300 (71.4)	399 380 (70.8)	102 697 (64.2)
Missing	411 206 (17.7)	118 021 (17.3)	39 021 (19.6)
First-degree family history of breast cancer			
No	1 817 368 (81.2)	572 979 (86.4)	167 006 (86.8)
Yes	420 085 (18.8)	89 920 (13.6)	25 292 (13.2)
Missing	82 563 (3.6)	19 084 (2.8)	6760 (3.4)
History of benign breast biopsy			
None (no prior biopsy)	1 774 790 (76.5)	569 618 (83.5)	168 766 (84.8)
Prior biopsy, benign diagnosis unknown	326 389 (14.1)	75 844 (11.1)	19 774 (9.9)
Nonproliferative	154 484 (6.7)	26 709 (3.9)	7764 (3.9)
Proliferative			
Without atypia	53 843 (2.3)	8574 (1.3)	2452 (1.2)
With atypia	10 510 (0.5)	1238 (0.2)	302 (0.2)
BI-RADS breast density			
Almost entirely fatty	223 242 (10.2)	66 257 (11.0)	20 113 (11.1)
Scattered fibroglandular densities	956 968 (43.5)	251 662 (41.9)	76 244 (42.2)
Heterogeneously dense	846 056 (38.5)	234 923 (39.1)	69 648 (38.6)
Extremely dense	171 555 (7.8)	47 545 (7.9)	14 465 (8.0)
Missing	122 195 (5.3)	81 596 (12.0)	18 588 (9.3)
BMI			
Underweight (<18.5)	25 413 (1.6)	8223 (1.6)	2135 (1.5)
Healthy weight (18.5-24.9)	688 504 (42.2)	206 268 (41.1)	53 582 (38.3)
Overweight (25.0-29.9)	474 728 (29.1)	142 810 (28.5)	39 749 (28.4)
Obesity			
Grade I (30.0-34.9)	253 933 (15.6)	78 469 (15.6)	23 457 (16.8)
Grade II/III (≥35.0)	188 333 (11.5)	65 716 (13.1)	20 980 (15.0)
Missing	689 105 (29.7)	180 497 (26.5)	59 155 (29.7)
Age at first live birth, y			
Nulliparous	386 859 (21.7)	115 875 (22.5)	31 956 (21.5)
<30	1 015 156 (57.0)	287 575 (55.8)	84 511 (57.0)
≥30	379 007 (21.3)	111 572 (21.7)	31 859 (21.5)
Missing	538 994 (23.2)	166 961 (24.5)	50 732 (25.5)
History of false-positive mammography results^c^			
No	1 806 747 (77.9)	584 748 (85.7)	176 064 (88.4)
Yes	513 269 (22.1)	97 235 (14.3)	22 994 (11.6)

Abbreviations: BI-RADS, Breast Imaging Reporting and Data System; BMI, body mass index (calculated as weight in kilograms divided by height in meters squared).

^a^ Among participants with nonmissing data.

^b^ Other includes American Indian or Alaska Native, Native Hawaiian or Pacific Islander, and self-reported other race.

^c^ False-positive screening mammography result within the previous 5 years.

In multivariable-adjusted analyses of a single screening round, DCIS detection was more likely with longer screening interval (biennial vs annual screening: odds ratio [OR], 1.43; 95% CI, 1.33-1.55; triennial vs annual screening: OR, 1.83; 95% CI, 1.63-2.05) (Table 2). Detection of DCIS was more common among women who had a first-degree family history of breast cancer, were nulliparous or 30 years or older at first live birth, had a prior benign breast biopsy, or reported Asian race (Table 2). Breast density was more strongly associated with DCIS detection among younger women, whereas prior false-positive mammography results were more strongly associated with DCIS detection among older women (Table 3). The positive association of BMI with DCIS detection was limited to postmenopausal women (Table 3). Detection of DCIS did not vary according to mammography modality (OR, 1.00; 95% CI, 0.89-1.12 for DBT vs digital mammography).

**Table 2.** DCIS Detection on a Single Screening Mammogram by Screening Interval and Selected Sociodemographic and Risk Factors

Characteristic	No. of screening mammograms	No. with screen-detected DCIS	DCIS detection rate per 1000 population	Multivariable-adjusted odds ratio (95% CI)^a^
Screening interval				
Annual	2 320 016	2474	1.07	1 [Reference]
Biennial	681 983	948	1.39	1.43 (1.33-1.55)
Triennial	199 058	335	1.68	1.83 (1.63-2.05)
First-degree family history of breast cancer				
No	2 557 353	2726	1.07	1 [Reference]
Yes	535 297	875	1.63	1.53 (1.42-1.65)
Age at first live birth, y				
Nulliparous	534 690	727	1.36	1.24 (1.14-1.36)
<30	1 387 242	1552	1.12	1 [Reference]
≥30	522 438	621	1.19	1.21 (1.11-1.33)
History of benign breast biopsy				
None (no prior biopsy)	2 513 174	2690	1.07	1 [Reference]
Prior biopsy, benign diagnosis unknown	422 007	633	1.50	1.26 (1.15-1.37)
Nonproliferative	188 957	269	1.42	1.24 (1.09-1.41)
Proliferative				
Without atypia	64 869	125	1.93	1.60 (1.33-1.92)
With atypia	12 050	40	3.32	2.66 (1.94-3.65)
Race and ethnicity				
Asian	369 730	555	1.50	1.37 (1.25-1.51)
Black	287 100	362	1.26	1.04 (0.93-1.17)
Hispanic/Latina	169 199	138	0.82	0.81 (0.68-0.96)
White	2 198 215	2505	1.14	1 [Reference]
Other or multiple races^b^	60 308	76	1.26	1.13 (0.89-1.42)

Abbreviation: DCIS, ductal carcinoma in situ.

^a^ Based on 20 imputed data sets. The multivariable model included screening interval, age (linear and squared), examination year (linear and squared), race and ethnicity, menopausal status, first-degree family history of breast cancer, personal history of breast biopsy, breast density, body mass index, age at first live birth, false-positive screening mammography result within the previous 5 years, interaction between linear age and breast density, interaction between age and false-positive screening mammography result within the previous 5 years, and interaction between menopausal status and body mass index.

^b^ Other included American Indian or Alaska Native, Native Hawaiian or Pacific Islander, and self-reported other race.

**Table 3.** DCIS Detection on a Single Screening Mammogram by Women’s Risk Factors That Interact With Age at Mammography or Menopausal Status

Characteristic	No. of screening mammograms	No. with screen-detected DCIS	DCIS detection rate per 1000 population	Multivariable-adjusted OR (95% CI)^a^			
				Age 40 y^b^	Age 50 y^b^	Age 60 y^b^	Age 70 y^b^
**BI-RADS breast density**							
Almost entirely fatty	309 612	186	0.60	0.38 (0.23-0.64)	0.44 (0.32-0.60)	0.49 (0.42-0.58)	0.56 (0.45-0.69)
Scattered fibroglandular densities	1 284 874	1374	1.07	1 [Reference]	1 [Reference]	1 [Reference]	1 [Reference]
Heterogeneously dense	1 150 627	1539	1.34	1.99 (1.64-2.42)	1.66 (1.47-1.86)	1.38 (1.27-1.49)	1.14 (1.01-1.29)
Extremely dense	233 565	332	1.42	2.35 (1.79-3.08)	1.90 (1.61-2.24)	1.53 (1.31-1.79)	1.24 (0.96-1.60)
**History of false-positive mammography results^c^**							
No	2 567 559	2804	1.09	1 [Reference]	1 [Reference]	1 [Reference]	1 [Reference]
Yes	633 498	953	1.50	1.08 (0.91-1.29)	1.23 (1.11-1.36)	1.39 (1.29-1.50)	1.58 (1.40-1.78)
**BMI^d^**							
Underweight (<18.5)	35 771	31	0.87	0.79 (0.50-1.24)		0.70 (0.48-1.00)	
Healthy weight (18.5-24.9)	948 354	1047	1.10	1 [Reference]		1 [Reference]	
Overweight (25.0-29.9)	657 287	721	1.10	1.01 (0.84-1.20)		1.23 (1.09-1.37)	
Obesity							
Grade I (30.0-34.9)	355 859	432	1.21	1.16 (0.90-1.49)		1.56 (1.36-1.78)	
Grade II/III (≥35.0)	275 029	318	1.16	1.18 (0.89-1.57)		1.72 (1.49-1.99)	

Abbreviations: BI-RADS, Breast Imaging Reporting and Data System; BMI, body mass index (calculated as weight in kilograms divided by height in meters squared); DCIS, ductal carcinoma in situ; OR, odds ratio.

^a^ Based on 20 imputed data sets. The multivariable model included screening interval, age (linear and squared), examination year (linear and squared), race and ethnicity, menopausal status, first-degree family history of breast cancer, personal history of breast biopsy, breast density, BMI, age at first live birth, false-positive screening mammography result within the previous 5 years, interaction between linear age and breast density, interaction between age and false-positive screening mammography result within the previous 5 years, and interaction between menopausal status and BMI.

^b^ Age was modeled as a continuous variable; ORs at specific decades of age are given to illustrate patterns in the interactions between age and other risk factors.

^c^ False-positive screening mammography result within the previous 5 years.

^d^ ORs under the columns for age 40 y and age 50 y indicate premenopausal; ORs under age 60 y and age 70 y indicate postmenopausal.

Overall, 11.2% of annual screeners had high 6-year risk of screen-detected DCIS compared with 2.7% among biennial screeners and 1.1% among triennial screeners (Table 4). Women aged 40 to 49 years had the lowest proportion in the intermediate or high-risk groups, whereas women aged 70 to 74 years had the highest proportion.

**Table 4.** Cumulative Risk of Screen-Detected Ductal Carcinoma In Situ After 6 Years of Annual, Biennial, or Triennial Screening^a^

Risk group	No. (%) of examinations by risk level				
	Very low (<0.10%)	Low (0.10%-0.19%)	Average (>0.19%-0.38%)	Intermediate (>0.38%-0.63%)	High (>0.63%)
**Annual**					
Overall	47 207 (1.5)	268 548 (8.4)	1 402 774 (43.8)	1 124 054 (35.1)	358 473 (11.2)
Age group, y					
40-49	37 948 (4.0)	156 068 (16.3)	529 250 (55.4)	211 452 (22.1)	21 383 (2.2)
50-59	9027 (0.9)	89 484 (8.7)	511 931 (49.7)	345 344 (33.5)	74 909 (7.3)
60-69	232 (0.0)	22 495 (2.5)	291 503 (32.6)	417 143 (46.6)	164 000 (18.3)
70-74	0 (0.0)	502 (0.2)	70 090 (22.0)	150 115 (47.1)	98 181 (30.8)
**Biennial**					
Overall	154 427 (4.8)	660 269 (20.6)	1 780 858 (55.6)	517 726 (16.2)	87 776 (2.7)
Age group, y					
40-49	102 094 (10.7)	308 176 (32.2)	495 955 (51.9)	47 178 (4.9)	2697 (0.3)
50-59	46 072 (4.5)	243 678 (23.6)	599 289 (58.1)	128 422 (12.5)	13 235 (1.3)
60-69	6213 (0.7)	96 257 (10.8)	527 984 (59.0)	224 549 (25.1)	40 372 (4.5)
70-74	48 (0.0)	12 158 (3.8)	157 631 (49.4)	117 578 (36.9)	31 472 (9.9)
**Triennial**					
Overall	279 219 (8.7)	992 054 (31.0)	1 618 357 (50.6)	277 519 (8.7)	33 909 (1.1)
Age group, y					
40-49	173 859 (18.2)	410 547 (42.9)	353 770 (37.0)	17 097 (1.8)	827 (0.1)
50-59	86 203 (8.4)	364 912 (35.4)	516 284 (50.1)	58 742 (5.7)	4555 (0.4)
60-69	18 743 (2.1)	189 491 (21.2)	543 585 (60.7)	128 494 (14.4)	15 060 (1.7)
70-74	414 (0.1)	27 104 (8.5)	204 718 (64.2)	73 185 (23.0)	13 467 (4.2)

^a^ The numbers (percentages) of screening examinations are adjusted by US population weights and standardized to same population for different screening intervals. High risk is the top 5%, intermediate risk is the 75th to 95th percentile, average risk is the 25th to 75th percentile, low risk is the 5th to 25th percentile, and very low risk is the lowest 5%.

The model predicting DCIS detection at a single screening round was well calibrated, with an E/O ratio of 1.00 (95% CI, 0.97-1.03) and little deviation from unity across all deciles of predicted risk (eFigure in Supplement 1). The adjusted AUC for predicting DCIS detection was 0.639 (95% CI, 0.630-0.648).

Mean cumulative 6-year risk of screen-detected DCIS was higher with increasing age and shorter screening interval (Figure; eTables 1-4 in Supplement 1). Among women aged 40 to 49 years, the mean 6-year screen-detected DCIS risk was 0.30% (IQR, 0.21%-0.37%) for annual screening, 0.21% (IQR, 0.14%-0.26%) for biennial screening, and 0.17% (IQR, 0.12%-0.22%) for triennial screening. For women aged 70 to 74 years, the mean cumulative risks were 0.58% (IQR, 0.41%-0.69%) after 6 annual screens, 0.40% (IQR, 0.28%-0.48%) after 3 biennial screens, and 0.33% (IQR, 0.23%-0.39%) after 2 triennial screens.

**Figure.**
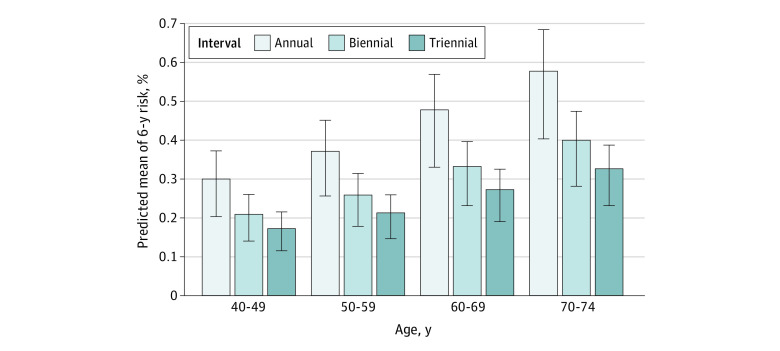
Mean Predicted Cumulative 6-Year Risk of Screen-Detected Ductal Carcinoma In Situ by Age and Screening Interval Within each age group, predictions were standardized to a common population for comparing predicted risks with different screening intervals. Weights of the study population were adjusted to reflect the US female population based on age, race and ethnicity, and first-degree family history of breast cancer. Error bars represent the IQRs.

eTables 1 through 4 in Supplement 1 list the mean cumulative 6-year risks of screen-detected DCIS by decade of age according to women’s risk factors and screening interval. For example, the 6-year risk of DCIS detection for women aged 50 to 59 years undergoing annual screening ranged from 0.34% (IQR, 0.24%-0.41%) for women with no prior benign breast biopsy to 1.11% (IQR, 0.80%-1.35%) for women with a history of proliferative benign breast disease with atypia, whereas the risk was 0.24% (IQR, 0.17%-0.29%) for women with no prior benign breast biopsy and 0.76% (IQR, 0.55%-0.93%) for women with a history of proliferative benign breast disease with atypia who underwent biennial screening.

## Discussion

The results of this cohort study suggest that DCIS detection rates on mammography screening vary by screening interval and clinical risk factors. Cumulative risk of screen-detected DCIS after 6 years of annual screening is substantially higher than for women undergoing 3 biennial screens. Age, first-degree family history of breast cancer, and history of benign breast biopsy are particularly strong risk factors for screen-detected DCIS. Breast density is a strong risk factor among younger women, and history of false-positive mammography results and obesity are strong risk factors among older women. Our risk prediction model integrates screening interval and individual risk factors to estimate the probability of screen-detected DCIS. These risk estimates can be used by policy makers in conjunction with estimates of other breast cancer screening outcomes (such as cumulative risk of false-positive mammography results and advanced cancer) when evaluating the balance of screening benefits and harms by screening interval.^15,16^

Ductal carcinoma in situ currently makes up more than 30% of screen-detected breast cancer in the US.^27^ Although the goal of breast cancer screening is early detection, screening recommendations from the US Preventive Services Task Force and the American Cancer Society acknowledge concerns about overdiagnosis and overtreatment of DCIS.^10,11^ Ductal carcinoma in situ is considered a nonobligate precursor of invasive breast cancer.^28^ Given the potential for subsequent invasive cancer and the current inability to reliably distinguish high-risk from indolent DCIS, treatment guidelines for DCIS recommend breast-conserving surgery and consideration of radiation therapy and endocrine therapy.^29^ Locoregional therapy reduces the risk of subsequent invasive breast cancer but has not been shown to influence overall survival or breast cancer–specific survival.^30,31,32,33,34,35^ Given the morbidity of DCIS treatments and evolving biological models of DCIS progression,^28^ many scientists have called for reconsideration of how DCIS is managed,^36,37,38^ and trials of active surveillance for low-grade DCIS are ongoing.^39,40,41^

Consistent with the recently published model of cumulative advanced breast cancer risk,^15^ we estimated 6-year risk of screen-detected DCIS to inform decision-making about mammography screening strategies. Previous studies^13,15,27,42^ have identified risk groups that can undergo biennial screening with little adverse change in risk of advanced cancer or life-years gained compared with annual mammography. Our results indicate that women who have low advanced cancer risk with biennial screening (eg, women with healthy weight and nondense breasts)^15^ would also experience reduced cumulative DCIS detection with a biennial vs annual screening interval. Of note, risk of screen-detected DCIS on a single screening round was higher with increasing time since last mammography, reflecting the longer interval for DCIS to emerge. However, the probability of screen-detected DCIS for biennial mammography is only 40% to 45% higher than annual mammography; similarly, the probability of screen-detected DCIS for triennial mammography is less than 3 times that of annual mammography. Consequently, cumulative DCIS risk after 6 years of screening is substantially lower for women undergoing 2 triennial or 3 biennial screens compared with 6 annual screens.

Our results do not directly provide new insights into the natural history of DCIS. Potential advantages of increased DCIS detection could include lower-interval invasive breast cancer rates.^5^ Annual screening may offer the opportunity to detect DCIS that has a short sojourn time.^43^ However, simulation modeling suggests that increased detection of DCIS with more frequent screening corresponds to increased overdiagnosis,^44^ and population-based data show that large increases in DCIS incidence do not lead to a reduction in early-stage invasive cancer incidence or mortality.^45^ Thus, uncertainty exists regarding whether screen-detected DCIS is a potential screening harm or benefit. Physicians referring women for screening may wish to consider advanced cancer risk as the primary outcome influencing screening frequency and supplemental imaging.^15^ Our results could be used to estimate the effect of the chosen screening strategy on the risk of DCIS detection and are relevant for policy makers considering a wide range of outcomes associated with different population-level screening strategies.^10^

Our study results are consistent with an extensive literature demonstrating that benign breast disease history, family history of breast cancer, breast density, BMI, and age at first live birth are associated with overall DCIS risk.^46,47,48,49^ To our knowledge, our study is the first to evaluate the history of false-positive mammography results in relation to future DCIS risk, although prior studies^50,51^ have identified false-positive mammography as a risk factor for breast cancer overall (invasive or DCIS). Our study provides new insights regarding interactions between age and breast density and false-positive mammography results in relation to risk of screen-detected DCIS. We also observed that the risk of screen-detected DCIS was higher among Asian women and lower among Hispanic/Latina women compared with White women. Reasons for these differences require further exploration.

Prior studies^52,53,54,55,56^ have demonstrated increases in overall or invasive breast cancer detection with DBT, but few have directly assessed DCIS detection. A meta-analysis^57^ of 4 European prospective, observational studies found that DCIS detection was higher on DBT vs digital mammography, whereas a large US-based observational study^58^ and a European randomized clinical trial^59^ both observed no difference in DCIS detection by modality. Our study found no difference in DCIS detection rate on DBT vs digital mammography after adjustment for other factors. Differences in study populations (eg, age and breast density), European vs US radiologist practices, the proportion of prevalent vs incident screening examinations, and covariate adjustments could contribute to the observed differences across studies.

### Strengths and Limitations

This study has several strengths, including the large, diverse, population-based sample and the prospective collection of risk factor information. However, as with any observational study, some limitations exist. Residual confounding could still impact differences in risk estimates by screening interval. Data on menopausal status and BMI were missing for a substantial fraction of examinations. We used multiple imputation to avoid bias that would have resulted from exclusion of examinations with incomplete data.^60^ We did not examine DCIS rates by nuclear grade, which correlates with risk of subsequent invasive breast cancer.^61^ We used cross-validation to assess the accuracy of our model. The AUC optimism and SEs for the risk factor ORs did not account for the process of selecting interactions for inclusion in the model and as a result may be underestimated. External validation is needed to evaluate model performance in other populations.^61^

## Conclusions

In summary, the results of this cohort study suggest wide variation in the probability of DCIS detection according to screening interval and clinical risk factors. Our risk model permits estimation of the probability of screen-detected DCIS during a 6-year time horizon according to mammography screening frequency and women’s risk factors. Our findings can be used by policy makers assessing the balance of benefits and harms of different screening strategies, in conjunction with existing risk models for other screening outcomes, such as advanced cancers and false-positive mammography results.^15,16^

## Notes

Supplement 1. **eMethods.** Technical Details of Risk Model Development and Evaluation **eFigure.** Calibration Results for Model Predicting Risk of Screen-Detected DCIS **eTable 1.** Variation in Mean Predicted Cumulative Six-Year Risk of Screen-Detected DCIS by Screening Interval and Risk Factors Among Women Aged 40-49 Years **eTable 2.** Variation in Mean Predicted Cumulative Six-Year Risk of Screen-Detected DCIS by Screening Interval and Risk Factors Among Women Aged 50-59 Years **eTable 3.** Variation in Mean Predicted Cumulative Six-Year Risk of Screen-Detected DCIS by Screening Interval and Risk Factors Among Women Aged 60-69 Years **eTable 4.** Variation in Mean Predicted Cumulative Six-Year Risk of Screen-Detected DCIS by Screening Interval and Risk Factors Among Women Aged 70-74 Years **eTable 5.** Summary of Variables in the Multiple Imputation Model

Supplement 2.
Data Sharing Statement
