# Supplementary material for: Cumulative 6-Year Risk of Screen-Detected Ductal Carcinoma In Situ by Screening Frequency
Source: JAMA Netw Open. 2023 Feb 20;6(2):e230166. doi: 10.1001/jamanetworkopen.2023.0166 (PMC9941892; doi:10.1001/jamanetworkopen.2023.0166)
Supplement: Supplement 1. — eMethods. Technical Details of Risk Model Development and Evaluation eFigure. Calibration Results for Model Predicting Risk of Screen-Detected DCIS eTable 1. Variation in Mean Predicted Cumulative Six-Year Risk of Screen-Detected DCIS by Screening Interval and Risk Factors Among Women Aged 40-49 Years eTable 2. Variation in Mean Predicted Cumulative Six-Year Risk of Screen-Detected DCIS by Screening Interval and Risk Factors Among Women Aged 50-59 Years eTable 3. Variation in Mean Predicted Cumulative Six-Year Risk of Screen-Detected DCIS by Screening Interval and Risk Factors Among Women Aged 60-69 Years eTable 4. Variation in Mean Predicted Cumulative Six-Year Risk of Screen-Detected DCIS by Screening Interval and Risk Factors Among Women Aged 70-74 Years eTable 5. Summary of Variables in the Multiple Imputation Model [file jamanetwopen-e230166-s001.pdf]

Supplementary Online Content

Sprague BL, Chen S, Miglioretti DL, et al. Cumulative 6-year risk of screen-detected ductal carcinoma in situ by screening frequency. *JAMA Netw Open*. 2023;6(2):e230166. doi:10.1001/jamanetworkopen.2023.0166

eMethods. Technical Details of Risk Model Development and Evaluation

eFigure. Calibration Results for Model Predicting Risk of Screen-Detected DCIS

eTable 1. Variation in Mean Predicted Cumulative Six-Year Risk of Screen-Detected DCIS by Screening Interval and Risk Factors Among Women Aged 40-49 Years

eTable 2. Variation in Mean Predicted Cumulative Six-Year Risk of Screen-Detected DCIS by Screening Interval and Risk Factors Among Women Aged 50-59 Years

eTable 3. Variation in Mean Predicted Cumulative Six-Year Risk of Screen-Detected DCIS by Screening Interval and Risk Factors Among Women Aged 60-69 Years

eTable 4. Variation in Mean Predicted Cumulative Six-Year Risk of Screen-Detected DCIS by Screening Interval and Risk Factors Among Women Aged 70-74 Years

eTable 5. Summary of Variables in the Multiple Imputation Model

This supplementary material has been provided by the authors to give readers additional information about their work.

© 2023 Sprague BL et al. *JAMA Network Open.*

eMethods. Technical details of risk model development and evaluation

All analyses were performed using the screening mammogram as the unit of analysis unless otherwise indicated. Data were analyzed using R version 4.0.4 (R Foundation for Statistical Computing, Vienna, Austria) and SAS version 9.4 (SAS Institute, Cary, NC). Two-sided alpha of 0.05 was used to determine statistical significance.

Multiple imputation

The primary outcome (screen-detected DCIS within 1 year follow-up of a positive screen) had no missing values. For modeling competing events (cancer diagnosis or death within 1, 2, or 3 years of an annual, biennial, or triennial screen, respectively), all annual screens had complete cancer capture for 1-year follow-up, while some biennial and triennial screens did not have complete capture for 2 and 3 years of follow-up, respectively. Therefore, some biennial and triennial screens had missing values for competing events. In addition, some covariates had missing data for some observations (Supplementary Table 5). Before model fitting, missing values were multiply imputed using MICE (multiple imputation chained equations), with $m = 20$ imputations. Supplementary eTable 5 summarizes the imputation model performed in SAS.

Model for risk of screen-detected DCIS

<u>Model fitting</u>: We used logistic regression to model the absolute risk of screen-detected DCIS as a function of age (linear and quadratic, centered at 55), year of screen (linear and quadratic, calculated based on the date of screen and centered at the latest time 01/31/2020 in the data), screening interval, mammography modality, menopausal status (premenopausal versus perimenopausal/postmenopausal), race/ethnicity, first-degree family history of breast cancer, history of benign biopsy, BMI category, breast density, age at first live birth (categorical), and prior false positive mammography. Fractional polynomials[1] were used to explore the presence of nonlinear relationships of the continuous predictor of age and year of screen, which showed that linear and quadratic terms would be adequate. Mammography modality was dropped due to non-significance. We evaluated interactions of risk factors with menopausal status, age and age squared within each imputed dataset. We retained those that were statistically significant at $p<0.05$ after combining across the imputed datasets; these included interaction between menopausal status and BMI, interaction between linear age and breast density, and interaction between linear age and prior false-positive mammography. Each of the multiply imputed datasets was used to obtain a separate fitted model, and estimated parameters (and standard errors) were combined to estimate odds ratios and confidence intervals for covariates using Rubin's rules.[3]

<u>Risk prediction and weight adjustment</u>: For each possible combination of covariates in the imputed datasets, the associated risk was predicted based on 20 fitted models from the multiply imputed datasets, giving 20 predicted risks. The final risk was the average of the 20 risks for each combination of covariates. To map back to the study population, a

© 2023 Sprague BL et al. *JAMA Network Open.*

weight was assigned to each covariate combination, which is the frequency of this covariate combination across all 20 imputed datasets. This base weight (w_0) was further adjusted to reflect the US population of women, by weighting based on age, race/ethnicity, and first degree family history of breast cancer: $w(age, race, family\ history, X)$= $w_0(age, race, family\ history, X) \times p_{US}(age, race) \times p_{US}(family\ history|age, race)$, where $p_{US}(age, race)$ is the proportion of women with this age and race in US population, $p_{US}(family\ history|age, race)$ is the proportion of women with or without family history within this age and race subgroup in US population, and X includes other covariates. The age and race/ethnicity distribution of the U.S. 2016 population was estimated from US census data.[4] The percentage of women with a first-degree family history by age and race/ethnicity was estimated from the 2015 NHIS.[5]

Evaluation of discriminatory accuracy: AUC was estimated using 5-fold cross-validation. Clustered randomization was used to ensure that all screens for a single woman were assigned to the same subset of the 5 cross-validation folds and that the same assignment was applied to all imputed datasets. The screening exams across 4 subsets were used as a training dataset to obtain fitted models (20 fitted models due to 20 imputed datasets) while the remaining 1 subset was used to obtain predicted risk and calculate AUC for validation. The multiple imputation process was performed for each cross-validation training dataset (i.e., the multiple imputation process was repeated 5 times). Predicted risk was estimated for each covariate and outcome combination in the validation subset and the final predicted risk was obtained as the average of the predicted risks based on the 20 imputed datasets. To map back to the validation population, a weight was assigned to each covariate and outcome combination as its proportion in this validation data (within each of the 20 imputed validation subsets) for AUC calculation, which led to 20 AUCs in each validation subset. The final AUC was the average of the 5×20 AUCs in 5 validation subsets.

To assess overfitting, we compared the AUC from the models fit using the full data (AUC_{All}) to AUCs from the models fit using above 5-fold cross-validation (AUC_{CV}). The overall risk evaluated was the average risk across the 20 imputed datasets. The estimated AUC_{All} was the average of 20 AUCs calculated using the overall risk to predict the outcomes in each of the 20 imputed datasets. The variance and the 95% confidence interval of the AUC_{All} were calculated using Rubin's Rules to account for the variance due to multiple imputation. The difference between them (AUC_{All} - AUC_{CV}) is called the optimism,[6] which is expected to be small if overfitting is small. To account for the overfitting, the adjusted AUC and its confidence interval were calculated by subtracting the optimism from AUC_{All} and its confidence limits:

$$AUC_{Adj} = AUC_{All} - \text{optimism} = AUC_{CV},$$

$$(CI_{Lower\ (Adj)}, CI_{Upper\ (Adj)}) = (CI_{Lower\ (All)} - \text{optimism}, CI_{Upper\ (All)} - \text{optimism}).$$

Model calibration: Model calibration was estimated by the ratio of expected to observed numbers of women with screen-detected DCIS, for all women and within risk decile subgroups, using 5-fold cross-validation. Similar to calculating AUC, the multiple imputation process was performed for each cross-validation training dataset. The predicted risk was estimated for each covariate combination in the validation subset and the (fold-specific) final predicted risk was

© 2023 Sprague BL et al. *JAMA Network Open.*

obtained as the average of the predicted risks based on the 20 imputed datasets in each validation subset. Combinations of covariates were classified into categories of low to high risk based on deciles of the predicted risks, where the predicted risk was calculated by the average of the fold-specific final predicted risks across the 5 folds. The expected-to-observed ratio (E/O) was calculated (overall and within each risk decile group) as the ratio of the average of the expected risk (E) to the observed proportion of cases (O). To map back to the validation population, a weight was assigned to each covariate and outcome combination as its proportion in the validation subset (within each of the 20 imputed validation subsets) for E and O calculation, which led to 20 Es and 20 Os in each validation subset. Within each validation subset, the 20 Es and 20 Os were combined using Rubin's Rule. The final E and O were their averages across the 5 folds. The observed risk was plotted against the expected risk for each decile risk group. For a decile risk group with expected proportion E, the Wald-type confidence interval was calculated for the observed proportion of cases within decile risk groups. Within each imputed dataset in a validation subset, the variance of observed proportion of cases was estimated as $O(1-O)/n$, where n is the 20% (to account for 1/5 sample size in validation subset) of total number of screens for overall population, or 20%×10% of the total sample size for each decile risk group. Within each fold, the 20 variances of observed proportion of cases were also combined using Rubin's Rule, and then the final variance was their average across the 5 folds and divided by 5 (to account for the increase in sample size when combining all 5 folds). The limits of confidence interval of E/O of overall or the decile risk group were then calculated by E divided by limits of confidence interval of O. Note that, the confidence intervals may be conservative (i.e., too narrow) due to ignoring variation between 5 cross-validation folds and variation in E estimates.

Model for risk of competing events

Like risk of screen-detected DCIS, we estimated the risk of competing events (death or invasive cancer) within one/two/three years after annual/biennial/triennial screening using logistic regression with the same study covariates. For the competing events, some biennial and triennial screens did not have complete capture for 2 and 3 years of follow-up, respectively. Therefore, some biennial and triennial screens had missing values for competing events and were included in imputation models to help impute missing study covariates (see Supplementary Table 5). However, for modeling the risk of competing events, we required complete capture following the screens (2 years for biennial and 3 years for triennial), and hence the screens without complete cancer capture were excluded in building the competing event risk model. For each possible combination of covariates, the final risk was the average of 20 risks based on fitted models of 20 imputed datasets.

Six-year cumulative risk of screen-detected DCIS

Estimate 6-year cumulative risk: The cumulative risk for screen-detected DCIS after six years of annual/biennial/triennial screening was calculated for each covariate combination, fixing the the year of screen at the latest date in the data

© 2023 Sprague BL et al. *JAMA Network Open.*

01/31/2020, based on the fitted logistic regression models. We used a discrete time survival model to estimate 6-year cumulative risk of screen-detected DCIS, while taking into account censoring and competing risks of other outcomes.[7] Let the covariates at the ith screen be denoted $X^{(i)}$ where *i* =1, …, 6 for annual screening, *i* =1, 2, 3 for biennial screening, and *i* =1, 2 for triennial screening. We assume that age increases by 1/2/3 years for each subsequent annual/biennial/triennial screen, *i.e.*, $age^{i} = age^{1} + k(i-1)$, ($k = 1, 2, 3$) while the other covariates remain the same as the first screen, i.e., $X^{(i)} = X^{(1)}$. The cumulative risks after six years of 6 annual screens were estimated by

$$risk^{ann} = \sum_{i=1}^{6}\left\{\left(1 - p_i^{comp(ann)}\right) p_i^{dcis(ann)} \prod_{j=1}^{i-1}(1 - p_j^{dcis(ann)})\right\}$$

Similarly, the cumulative risks after six years of 3 biennial screens were estimated by

$$risk^{bie} = \sum_{i=1}^{3}\left\{\left(1 - p_i^{comp(bie)}\right) p_i^{dcis(bie)} \prod_{j=1}^{i-1}(1 - p_j^{dcis(bie)})\right\},$$

and the cumulative risks after six years of 2 triennial screens were estimated by

$$risk^{tri} = \sum_{i=1}^{2}\left\{\left(1 - p_i^{comp(tri)}\right) p_i^{dcis(bie)} \prod_{j=1}^{i-1}(1 - p_j^{dcis(tri)})\right\},$$

where $p_i^{dcis(ann)}$ (or $p_i^{dcis(bie)},\ p_i^{dcis(tri)}$) is the predicted risk of screen-detected DCIS after an annual/biennial/triennial screen given covariates $X^{(i)}$, $p_i^{comp(ann)}$ (or $p_i^{comp(bie)},\ p_i^{comp(tri)}$) is the predicted risk of competing events within 1/2/3 years after an annual/biennial/triennial screen given covariates $X^{(i)}$, and $\prod_{j=1}^{i-1}(1 - p_j^{dcis(ann)}) = \prod_{j=1}^{i-1}(1 - p_j^{dcis(bie)}) = \prod_{j=1}^{i-1}(1 - p_j^{dcis(tri)}) = 1$ when $i = 1$.

For each possible combination of covariates, 20 cumulative risks were estimated based on 20 fitted models using 20 imputed datasets and averaged to estimate the cumulative risk for that covariate combination.

<u>Comparing screening intervals regarding 6-year cumulative risk</u>: To compare screening intervals within the same population, 6-year cumulative risks with different intervals were estimated for each covariate combination, fixing the year of screen at the latest date in the data 01/31/2020. Like the previous section on weight adjustment, weights were assigned to covariate combinations to reflect the US population of women. For each subgroup of woman-level characteristics, the predicted risks from all possible covariate combinations in that subgroup were pooled, and the (weighted) mean 6-year cumulative risks and interquartile ranges (IQR) were calculated based on the pooled cumulative risks.

© 2023 Sprague BL et al. *JAMA Network Open.*

References

1. Sauerbrei W, Royston P, Binder H: Selection of important variables and determination of functional form for continuous predictors in multivariable model building. *Stat Med* 2007;26:5512-28.

2. Raghunathan, T. E. and Dong, Q. (2011). "Analysis of variance from multiply imputed data sets." Unpublished manuscript. Survey Research Center, Institute for Social Research, University of Michigan, Ann Arbor, Michigan. Retrieved from http://www-personal.umich.edu/~teraghu/Raghunathan-Dong.pdf.

3. Marshall A, Altman DG, Holder RL, Royston P: Combining estimates of interest in prognostic modelling studies after multiple imputation: Current practice and guidelines. *BMC Medical Research Methodology* 2009;9:57.

4. Annual estimates of the resident population by sex, single year of age, race, and Hispanic origin for the United States: April 1, 2010 to July 1, 2016. U.S. Census Bureau, Population Division; June 2017. https://www.census.gov/programs-surveys/popest/data/tables.2017.html. Accessed 5/27/2019.

5. 2015 National Health Interview Survey (NHIS). National Center for Health Statistics. https://nhis.ipums.org/nhis/resources/srvydesc2015.pdf. Accessed Jan. 25, 2022.

6. Steyerberg EW, Harrell FE, Borsboom GJJ, Eijkemans MJ, Vergouwe Y, Habbema JDF: Internal validation of predictive models. *J Clin Epidemiol* 2001;54:774–781.

7. Hubbard RA, Ripping TM, Chubak J, Broeders MJ, Miglioretti DL: Statistical methods for estimating the cumulative risk of screening mammography outcomes. *Cancer Epidemiol Biomarkers Prev* 2016;25:513-20.

© 2023 Sprague BL et al. *JAMA Network Open.*

eFigure. Calibration results for model predicting risk of screen-detected DCIS.

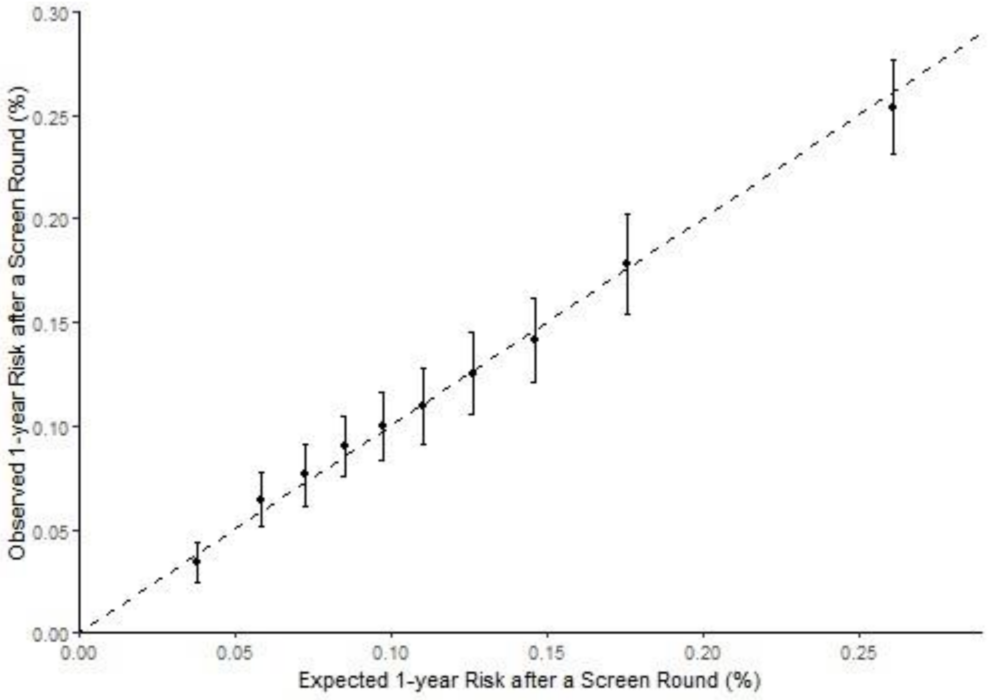

Decile	Expected 1-year rate after a round (E) (%)	Observed 1-year rate after a round (O) (%)	All screens E/O (95% CI)
1	0.038	0.034	1.12 (0.88 to 1.55)
2	0.059	0.064	0.91 (0.76 to 1.15)
3	0.073	0.077	0.95 (0.80 to 1.18)
4	0.085	0.090	0.94 (0.81 to 1.13)
5	0.098	0.100	0.97 (0.84 to 1.16)
6	0.111	0.109	1.01 (0.87 to 1.22)
7	0.126	0.125	1.01 (0.87 to 1.20)
8	0.146	0.141	1.03 (0.90 to 1.21)
9	0.176	0.178	0.99 (0.87 to 1.14)
10	0.261	0.254	1.03 (0.94 to 1.13)
Overall	0.117	0.117	1.00 (0.97 to 1.03)

© 2023 Sprague BL et al. *JAMA Network Open.*

eTable 1. Variation in mean predicted cumulative six-year risk of screen-detected DCIS by screening interval and risk factors among women aged 40-49 years. Within this age group, a standardized population is used for comparing predicted risks across screening interval. The weights of the study population were adjusted to reflect the US female population in this age group based on age, race/ethnicity, and first-degree family history of breast cancer.

	Mean predicted cumulative six-year risk of screen-detected DCIS, % (interquartile range)		
Characteristic	Annual	Biennial	Triennial
**Overall**	0.30 (0.21-0.37)	0.21 (0.14-0.26)	0.17 (0.12-0.22)
**Menopausal Status**			
Premenopausal	0.31 (0.21-0.38)	0.21 (0.15-0.26)	0.18 (0.12-0.22)
Postmenopausal	0.26 (0.18-0.32)	0.18 (0.12-0.22)	0.15 (0.10-0.18)
**First-degree family history of breast cancer**			
No	0.29 (0.20-0.36)	0.20 (0.14-0.25)	0.17 (0.11-0.21)
Yes	0.46 (0.32-0.57)	0.32 (0.22-0.40)	0.26 (0.19-0.33)
**History of benign breast biopsy**			
None (no prior biopsy)	0.28 (0.20-0.35)	0.20 (0.14-0.25)	0.16 (0.11-0.21)
Prior biopsy, benign diagnosis unknown	0.39 (0.29-0.48)	0.27 (0.20-0.33)	0.22 (0.17-0.27)
Non-proliferative	0.39 (0.28-0.48)	0.27 (0.20-0.34)	0.23 (0.16-0.28)
Proliferative without atypia	0.55 (0.40-0.67)	0.38 (0.28-0.47)	0.31 (0.23-0.38)
Proliferative with atypia	0.93 (0.69, 1.12)	0.65 (0.48-0.78)	0.53 (0.39-0.64)
**BI-RADS breast density**			
Almost entirely fatty	0.09 (0.07-0.11)	0.06 (0.05-0.07)	0.05 (0.04-0.06)
Scattered fibroglandular densities	0.21 (0.16-0.24)	0.14 (0.11-0.17)	0.12 (0.09-0.14)
Heterogeneously dense	0.36 (0.28-0.40)	0.25 (0.20-0.28)	0.21 (0.16-0.23)
Extremely dense	0.41 (0.33-0.47)	0.29 (0.23-0.33)	0.24 (0.19-0.27)
**Body mass index, kg/m^2**			
Underweight (<18.5)	0.30 (0.22-0.35)	0.21 (0.16-0.25)	0.17 (0.13-0.20)
Normal (18.5-24.9)	0.34 (0.25-0.40)	0.23 (0.17-0.28)	0.19 (0.14-0.23)
Overweight (25.0-29.9)	0.28 (0.19-0.35)	0.20 (0.13-0.24)	0.16 (0.11-0.20)
Obese I (30.0-34.9)	0.29 (0.19-0.36)	0.20 (0.13-0.25)	0.16 (0.11-0.21)
Obese II/III (≥35.0)	0.25 (0.15-0.32)	0.17 (0.11-0.23)	0.14 (0.09-0.19)
**Age at first live birth**			
Nulliparous	0.35 (0.24-0.42)	0.24 (0.17-0.30)	0.20 (0.14-0.25)
Age < 30 years	0.26 (0.18-0.32)	0.18 (0.13-0.23)	0.15 (0.10-0.19)
Age ≥ 30 years	0.34 (0.24-0.41)	0.24 (0.17-0.29)	0.19 (0.14-0.24)
**History of false-positive mammography**[a]			
No	0.28 (0.19-0.35)	0.20 (0.14-0.25)	0.16 (0.11-0.21)
Yes	0.37 (0.25-0.45)	0.25 (0.18-0.31)	0.21 (0.14-0.26)
**Race/ethnicity**			

© 2023 Sprague BL et al. *JAMA Network Open.*

Asian	0.44 (0.36-0.51)	0.31 (0.25-0.36)	0.26 (0.21-0.30)
Black	0.30 (0.21-0.37)	0.21 (0.14-0.26)	0.17 (0.12-0.21)
Hispanic/Latina	0.23 (0.16-0.28)	0.16 (0.11-0.20)	0.13 (0.09-0.16)
White	0.31 (0.21-0.37)	0.21 (0.15-0.26)	0.18 (0.12-0.22)
Other/Multiple	0.34 (0.24-0.42)	0.24 (0.17-0.29)	0.20 (0.14-0.24)

BI-RADS, Breast Imaging Reporting and Data System.

[a]False-positive screening mammogram within prior 5 years.

© 2023 Sprague BL et al. *JAMA Network Open.*

eTable 2. Variation in mean predicted cumulative six-year risk of screen-detected DCIS by screening interval and risk factors among women aged 50-59 years. Within this age group, a standardized population is used for comparing predicted risks across screening interval. The weights of the study population were adjusted to reflect the US female population in this age group based on age, race/ethnicity, and first-degree family history of breast cancer.

	Mean predicted cumulative six-year risk of screen-detected DCIS, % (interquartile range)		
Characteristic	Annual	Biennial	Triennial
**Overall**	0.37 (0.26-0.45)	0.26 (0.18-0.32)	0.21 (0.15-0.26)
**Menopausal Status**			
Premenopausal	0.42 (0.29-0.50)	0.29 (0.20-0.35)	0.24 (0.17-0.29)
Postmenopausal	0.36 (0.25-0.44)	0.25 (0.17-0.31)	0.21 (0.14-0.25)
**First-degree family history of breast cancer**			
No	0.35 (0.25-0.42)	0.24 (0.18-0.30)	0.20 (0.14-0.25)
Yes	0.55 (0.40-0.67)	0.39 (0.28-0.47)	0.32 (0.23-0.39)
**History of benign breast biopsy**			
None (no prior biopsy)	0.34 (0.24-0.41)	0.24 (0.17-0.29)	0.20 (0.14-0.24)
Prior biopsy, benign diagnosis unknown	0.46 (0.34-0.56)	0.32 (0.23-0.39)	0.26 (0.19-0.32)
Non-proliferative	0.48 (0.35-0.59)	0.33 (0.24-0.41)	0.27 (0.20-0.34)
Proliferative without atypia	0.65 (0.47-0.79)	0.45 (0.33-0.55)	0.37 (0.27-0.45)
Proliferative with atypia	1.11 (0.80, 1.35)	0.76 (0.55-0.93)	0.62 (0.45-0.76)
**BI-RADS breast density**			
Almost entirely fatty	0.17 (0.13-0.20)	0.12 (0.09-0.14)	0.10 (0.07-0.11)
Scattered fibroglandular densities	0.33 (0.24-0.38)	0.23 (0.17-0.27)	0.19 (0.14-0.22)
Heterogeneously dense	0.45 (0.34-0.53)	0.32 (0.24-0.37)	0.26 (0.20-0.30)
Extremely dense	0.49 (0.37-0.57)	0.34 (0.26-0.40)	0.29 (0.22-0.33)
**Body mass index, kg/m^2**			
Underweight (<18.5)	0.31 (0.22-0.37)	0.21 (0.15-0.26)	0.18 (0.13-0.21)
Normal (18.5-24.9)	0.37 (0.26-0.44)	0.26 (0.18-0.31)	0.21 (0.15-0.26)
Overweight (25.0-29.9)	0.36 (0.25-0.44)	0.25 (0.18-0.31)	0.21 (0.15-0.25)
Obese I (30.0-34.9)	0.40 (0.28-0.49)	0.28 (0.20-0.34)	0.23 (0.16-0.28)
Obese II/III (≥35.0)	0.38 (0.23-0.48)	0.26 (0.16-0.33)	0.22 (0.13-0.27)
**Age at first live birth**			
Nulliparous	0.43 (0.31-0.52)	0.30 (0.21-0.36)	0.25 (0.18-0.30)
Age < 30 years	0.34 (0.24-0.41)	0.23 (0.16-0.28)	0.19 (0.14-0.23)
Age ≥ 30 years	0.41 (0.29-0.50)	0.29 (0.21-0.35)	0.24 (0.17-0.29)
**History of false-positive mammography**[a]			
No	0.34 (0.24-0.41)	0.24 (0.17-0.29)	0.20 (0.14-0.24)
Yes	0.50 (0.35-0.60)	0.35 (0.24-0.42)	0.28 (0.20-0.34)
**Race/ethnicity**			

© 2023 Sprague BL et al. *JAMA Network Open.*

Asian	0.49 (0.37-0.57)	0.34 (0.26-0.40)	0.28 (0.21-0.33)
Black	0.40 (0.28-0.48)	0.28 (0.20-0.33)	0.23 (0.16-0.27)
Hispanic/Latina	0.28 (0.20-0.34)	0.20 (0.14-0.24)	0.16 (0.11-0.20)
White	0.38 (0.26-0.45)	0.26 (0.18-0.32)	0.22 (0.15-0.26)
Other/Multiple	0.41 (0.29-0.49)	0.28 (0.20-0.34)	0.23 (0.17-0.28)

BI-RADS, Breast Imaging Reporting and Data System.

[a]False-positive screening mammogram within 5 years prior to index mammogram.

© 2023 Sprague BL et al. *JAMA Network Open.*

eTable 3. Variation in mean predicted cumulative six-year risk of screen-detected DCIS by screening interval and risk factors among women aged 60-69 years. Within this age group, a standardized population is used for comparing predicted risks across screening interval. The weights of the study population were adjusted to reflect the US female population in this age group based on age, race/ethnicity, and first-degree family history of breast cancer.

	Mean predicted cumulative six-year risk of screen-detected DCIS, % (interquartile range)		
Characteristic	Annual	Biennial	Triennial
**Overall**	0.48 (0.33-0.57)	0.33 (0.23-0.40)	0.27 (0.19-0.33)
**First-degree family history of breast cancer**			
No	0.44 (0.32-0.52)	0.31 (0.22-0.37)	0.25 (0.18-0.30)
Yes	0.69 (0.50-0.81)	0.48 (0.35-0.57)	0.39 (0.29-0.46)
**History of benign breast biopsy**			
None (no prior biopsy)	0.43 (0.31-0.52)	0.30 (0.22-0.36)	0.25 (0.18-0.30)
Prior biopsy, benign diagnosis unknown	0.58 (0.42-0.69)	0.40 (0.29-0.48)	0.33 (0.24-0.39)
Non-proliferative	0.62 (0.44-0.75)	0.43 (0.31-0.52)	0.35 (0.25-0.43)
Proliferative without atypia	0.83 (0.59, 1.00)	0.57 (0.41-0.69)	0.47 (0.33-0.56)
Proliferative with atypia	1.42 (1.01, 1.71)	0.97 (0.70, 1.17)	0.78 (0.57-0.94)
**BI-RADS breast density**			
Almost entirely fatty	0.28 (0.21-0.32)	0.19 (0.14-0.22)	0.16 (0.12-0.18)
Scattered fibroglandular densities	0.48 (0.35-0.56)	0.33 (0.24-0.39)	0.27 (0.20-0.32)
Heterogeneously dense	0.56 (0.40-0.65)	0.39 (0.28-0.45)	0.32 (0.24-0.37)
Extremely dense	0.55 (0.41-0.65)	0.39 (0.29-0.46)	0.32 (0.24-0.38)
**Body mass index, kg/m^2**			
Underweight (<18.5)	0.34 (0.25-0.40)	0.23 (0.18-0.28)	0.19 (0.14-0.23)
Normal (18.5-24.9)	0.43 (0.32-0.51)	0.30 (0.23-0.36)	0.25 (0.19-0.29)
Overweight (25.0-29.9)	0.47 (0.35-0.56)	0.33 (0.24-0.39)	0.27 (0.20-0.32)
Obese I (30.0-34.9)	0.55 (0.39-0.66)	0.38 (0.28-0.46)	0.31 (0.23-0.38)
Obese II/III (≥35.0)	0.55 (0.34-0.67)	0.38 (0.24-0.46)	0.31 (0.19-0.38)
**Age at first live birth**			
Nulliparous	0.55 (0.39-0.66)	0.38 (0.27-0.46)	0.32 (0.23-0.38)
Age < 30 years	0.45 (0.31-0.53)	0.31 (0.22-0.37)	0.26 (0.18-0.31)
Age ≥ 30 years	0.52 (0.38-0.62)	0.36 (0.26-0.43)	0.30 (0.22-0.35)
**History of false-positive mammography**[a]			
No	0.43 (0.32-0.52)	0.30 (0.22-0.36)	0.25 (0.18-0.30)
Yes	0.71 (0.51-0.84)	0.49 (0.36-0.58)	0.40 (0.29-0.48)
**Race/ethnicity**			
Asian	0.56 (0.43-0.64)	0.39 (0.30-0.45)	0.33 (0.25-0.37)
Black	0.52 (0.38-0.61)	0.36 (0.27-0.43)	0.30 (0.22-0.35)

© 2023 Sprague BL et al. *JAMA Network Open.*

Hispanic/Latina	0.36 (0.26-0.42)	0.25 (0.18-0.30)	0.21 (0.15-0.25)
White	0.48 (0.34-0.57)	0.34 (0.23-0.40)	0.28 (0.19-0.33)
Other/Multiple	0.51 (0.36-0.61)	0.36 (0.25-0.42)	0.29 (0.21-0.35)

BI-RADS, Breast Imaging Reporting and Data System.

[a]False-positive screening mammogram within prior 5 years.

© 2023 Sprague BL et al. *JAMA Network Open.*

eTable 4. Variation in mean predicted cumulative six-year risk of screen-detected DCIS by screening interval and risk factors among women aged 70-74 years. Within this age group, a standardized population is used for comparing predicted risks across screening interval. The weights of the study population were adjusted to reflect the US population in this age group based on age, race/ethnicity, and first-degree family history of breast cancer.

	Mean predicted cumulative six-year risk of screen-detected DCIS, % (interquartile range)		
Characteristic	Annual	Biennial	Triennial
**Overall**	0.58 (0.41-0.69)	0.40 (0.28-0.48)	0.33 (0.23-0.39)
**First-degree family history of breast cancer**			
No family history	0.53 (0.37-0.61)	0.37 (0.26-0.43)	0.30 (0.22-0.35)
Family History	0.83 (0.60-0.96)	0.57 (0.42-0.67)	0.47 (0.34-0.54)
**History of benign breast biopsy**			
None (no prior biopsy)	0.52 (0.37-0.61)	0.36 (0.26-0.42)	0.30 (0.21-0.35)
Prior biopsy, benign diagnosis unknown	0.68 (0.47-0.79)	0.47 (0.33-0.55)	0.38 (0.27-0.45)
Non-proliferative	0.75 (0.52-0.90)	0.52 (0.36-0.63)	0.42 (0.29-0.51)
Proliferative without atypia	1.02 (0.70, 1.22)	0.70 (0.49-0.84)	0.56 (0.40-0.68)
Proliferative with atypia	1.70 (1.16, 2.05)	1.15 (0.80, 1.39)	0.91 (0.65, 1.11)
**BI-RADS breast density**			
Almost entirely fatty	0.38 (0.27-0.43)	0.26 (0.19-0.30)	0.21 (0.16-0.24)
Scattered fibroglandular densities	0.61 (0.43-0.70)	0.42 (0.30-0.49)	0.34 (0.25-0.40)
Heterogeneously dense	0.63 (0.45-0.72)	0.44 (0.32-0.50)	0.36 (0.26-0.41)
Extremely dense	0.61 (0.41-0.73)	0.42 (0.29-0.51)	0.35 (0.24-0.42)
**Body mass index, kg/m^2**			
Underweight (<18.5)	0.39 (0.28-0.44)	0.27 (0.20-0.31)	0.22 (0.16-0.25)
Normal (18.5-24.9)	0.50 (0.37-0.58)	0.35 (0.26-0.41)	0.29 (0.21-0.33)
Overweight (25.0-29.9)	0.57 (0.43-0.68)	0.40 (0.30-0.47)	0.32 (0.25-0.39)
Obese I (30.0-34.9)	0.69 (0.52-0.83)	0.47 (0.36-0.57)	0.39 (0.29-0.47)
Obese II/III (≥35.0)	0.70 (0.46-0.82)	0.48 (0.32-0.56)	0.39 (0.26-0.45)
**Age at first live birth**			
Nulliparous	0.68 (0.46-0.79)	0.47 (0.32-0.55)	0.38 (0.27-0.45)
Age < 30 years	0.55 (0.37-0.64)	0.38 (0.26-0.45)	0.31 (0.22-0.37)
Age ≥ 30 years	0.65 (0.45-0.75)	0.45 (0.31-0.52)	0.37 (0.26-0.43)
**History of false-positive mammography**[a]			
No	0.51 (0.37-0.59)	0.35 (0.26-0.41)	0.29 (0.22-0.34)
Yes	0.91 (0.67, 1.07)	0.63 (0.46-0.73)	0.51 (0.38-0.59)
**Race/ethnicity**			
Asian	0.70 (0.51-0.79)	0.49 (0.36-0.55)	0.40 (0.30-0.45)
Black	0.66 (0.46-0.77)	0.46 (0.32-0.53)	0.37 (0.27-0.43)

© 2023 Sprague BL et al. *JAMA Network Open.*

Hispanic/Latina	0.45 (0.33-0.53)	0.31 (0.23-0.37)	0.26 (0.19-0.30)
White	0.57 (0.41-0.68)	0.40 (0.28-0.47)	0.33 (0.23-0.39)
Other/Multiple	0.64 (0.45-0.75)	0.44 (0.31-0.51)	0.36 (0.26-0.42)

BI-RADS, Breast Imaging Reporting and Data System; DBT, digital breast tomosynthesis; DM, digital mammography.
[a]False-positive screening mammogram within prior 5 years

© 2023 Sprague BL et al. *JAMA Network Open.*

eTable 5. Summary of variables in the multiple imputation model.[a]

Description	Type	FCS	Total N = 3,201,057
			Missing N (%)
Screening interval	Binary	N/A	0 (0)
Screen-detected DCIS within 1 year follow-up	Binary	N/A	0 (0)
Invasive cancer diagnosed in 1-year follow-up	Binary	N/A	0 (0)
Invasive cancer diagnosed in 2-year follow-up	Binary	N/A	0 (0)
Invasive cancer diagnosed in 3-year follow-up	Binary	N/A	0 (0)
BCSC registry site	Nominal	N/A	0 (0)
Facility ID (not used for imputing density)	Nominal	N/A	0 (0)
Reader ID (only used for imputing density)	Nominal	N/A	0 (0)
Prior false positive mammogram	Binary	N/A	0 (0)
Age (linear and quadratic) at exam	Continuous	N/A	0 (0)
Calendar year of exam (linear and quadratic)	Continuous	N/A	0 (0)
Most severe benign biopsy result	Nominal	N/A	0 (0)
Mammogram modality	Binary	N/A	0 (0)
Final mammogram result	Binary	N/A	0 (0)
Initial mammogram result	Binary	N/A	2 (0.0)
First-degree family history of breast cancer	Binary	discrim	108,407 (3.4)
Race/ethnicity	Nominal	discrim	116,505 (3.6)
BI-RADS breast density category	Nominal	discrim	222,379 (7.0)
Competing event[b]	Binary	discrim	408,477 (12.8)

© 2023 Sprague BL et al. *JAMA Network Open.*

Menopausal status	Binary	discrim	568,248 (17.8)
Age at first birth	Nominal	discrim	756,687 (23.6)
Body Mass Index (BMI) category	Ordinal	discrim	928,757 (29.0)

[a]FCS, type of Fully Conditional Specification statement used in SAS PROC MI; BI-RADS, Breast Imaging Reporting and Data System; discrim, FCS statement option in SAS PROC MI to impute categorical variable; N/A, no missing value and hence imputation not needed; BCSC, Breast Cancer Surveillance Consortium.

[b]Competing events included invasive cancer diagnosis or death.

© 2023 Sprague BL et al. *JAMA Network Open.*
