# Supplementary material for: Cumulative 6-Year Risk of Screen-Detected Ductal Carcinoma In Situ by Screening Frequency
Source: JAMA Netw Open. 2023 Feb 20;6(2):e230166. doi: 10.1001/jamanetworkopen.2023.0166 (PMC9941892; doi:10.1001/jamanetworkopen.2023.0166)
Supplement: Supplement 2. — Data Sharing Statement [file jamanetwopen-e230166-s002.pdf]

Data Sharing Statement

Sprague. Cumulative 6-Year Risk of Screen-Detected Ductal Carcinoma In Situ by Screening Frequency. *JAMA Netw Open*. Published February 20, 2023. doi:10.1001/jamanetworkopen.2023.0166

Data
Data available: Yes
Data types: Deidentified participant data
How to access data: Deidentified data underlying this manuscript is available, upon reasonable request and with appropriate regularly approvals, from the Breast Cancer Surveillance Consortium Statistical Coordinating Center (KPWA.scc@kp.org).
When available: With publication

Supporting Documents
Document types: None

Additional Information
Who can access the data: Researchers whose proposed use of the data has been approved.
Types of analyses: For non-commercial applications.
Mechanisms of data availability: With support to cover costs of data preparation and with appropriately regulatory approvals for data disclosure.
